# C-X-C motif chemokine receptor 4-directed PET signal in the arterial tree is not consistently linked to calcified plaque burden and cardiovascular risk

**DOI:** 10.7150/thno.102910

**Published:** 2025-01-01

**Authors:** Aleksander Kosmala, Natalie Hasenauer, Sebastian E. Serfling, Kerstin Michalski, Matthias Fröhlich, Niklas Dreher, Philipp E. Hartrampf, Takahiro Higuchi, Andreas K. Buck, Alexander Weich, Theresa Reiter, Rudolf A. Werner

**Affiliations:** 1 Department of Nuclear Medicine, University Hospital Würzburg, Würzburg, Germany.; 2 Internal Medicine II, University Hospital Würzburg, Würzburg, Germany.; 3 Nuclear Medicine, Faculty of Medicine, University of Augsburg, Augsburg, Germany.; 4 Faculty of Medicine, Dentistry and Pharmaceutical Sciences, Okayama University, Okayama, Japan.; 5 NET-Zentrum Würzburg, European Neuroendocrine Tumor Society Center of Excellence (ENETS CoE), University Hospital Würzburg, Würzburg, Germany.; 6 Internal Medicine I, University Hospital Würzburg, Würzburg, Germany.; 7 Department of Electrophysiology, German Heart Center Munich, Technical University of Munich, Munich, Germany.; 8 Johns Hopkins School of Medicine, The Russell H Morgan Department of Radiology and Radiological Sciences, Baltimore, MD, United States.; 9 Goethe University Frankfurt, University Hospital, Clinic for Diagnostic and Interventional Radiology and Nuclear Medicine, Department of Nuclear Medicine, Germany.

**Keywords:** [^68^Ga]Ga-PentixaFor, C-X-C motif chemokine receptor 4, CXCR4, atherosclerosis, cardiovascular risk factors, molecular imaging

## Abstract

**Purpose:** To establish the extent, distribution and frequency of in-vivo vessel wall [^68^Ga]Ga-PentixaFor uptake and to determine its relationship with calcified atherosclerotic plaque burden (CAP) and cardiovascular risk factors (CVRF).

**Methods:** 65 oncological patients undergoing [^68^Ga]Ga-PentixaFor PET/CT were assessed. Radiotracer uptake (target-to-background ratio [TBR]) and CAP burden (including number of CAP sites, calcification circumference and thickness) in seven major vessel segments per patient were determined. We then investigated associations of vessel wall uptake with CAP burden, cardiovascular risk (CVRF and European Society of Cardiology [ESC] SCORE2/SCORE2-OP risk chart) and image noise (determined by coefficient of variation [CoV] from unaffected liver parenchyma).

**Results:** We identified 1292 sites of high focal [^68^Ga]Ga-PentixaFor uptake (PentixaFor+ sites) in the vessel wall in 65/65 (100%) patients, with concomitant calcification in 385/1292 (29.8%) sites. There were no significant associations between vessel wall uptake and CAP burden (number of PentixaFor+ sites: r ≤ 0.18, *P* ≥ 0.14; PentixaFor+ TBR: r ≤ 0.08, *P* ≥ 0.54). The number of PentixaFor+ sites showed a moderate correlation with cardiovascular risk (ESC SCORE2/SCORE2-OP, r = 0.30; number of CVRF, r = 0.26; *P* = 0.04, respectively), but failed to reach significance for PentixaFor+ TBR (r ≤ 0.18, *P* ≥ 0.22). In univariable regression analysis, body mass index (odds ratio [OR] 1.08, 95%-confidence interval [CI] 1.02-1.14) and CoV (OR, 1.07; CI, 1.05-1.10) were linked to TBR and the number of PentixaFor+ sites (*P* < 0.01, respectively), while injected activity was only associated with the latter imaging parameter (OR, 0.99; CI, 0.98-1.00; *P* = 0.04). In multivariable regression, injected activity (OR, 1.00; CI, 0.99-1.00) and CoV (OR, 1.06; CI, 1.06-1.07) remained significantly associated with the number of PentixaFor+ sites (*P* < 0.01, respectively). CoV, however, was the only parameter significantly linked to PentixaFor+ TBR on multivariable analysis (OR, 1.02; CI, 1.01-1.03; *P* < 0.01).

**Conclusion:** On a visual and quantitative level, high focal [^68^Ga]Ga-PentixaFor uptake in the arterial tree was not consistently linked to vessel wall calcification or cardiovascular risk. Image noise, however, may account for a substantial portion of apparent vessel wall uptake.

## Introduction

The C-X-C motif chemokine receptor 4 (CXCR4) plays a critical role in cancer progression by facilitating tumor spread through angiogenesis, cell growth and immune response inhibition 1-6. The ^68^Ga-labelled, CXCR4-directed radiotracer PentixaFor has been used in clinical oncology, particularly in patients with hematologic malignancies and solid tumors such as lung cancer, neuroendocrine neoplasms and adrenocortical carcinoma 6-11. CXCR4 has also been advocated to play a crucial role in atherosclerosis by mobilizing and recruiting progenitor and inflammatory cells 12. However, with conflicting evidence regarding its potential role in promoting or protecting against atherosclerosis progression, the specific function of CXCR4 and its ligands C-X-C motif chemokine 12 and migration inhibitory factor remains incompletely understood 12, 13.

PET agents including CXCR4-directed [^68^Ga]Ga-PentixaFor have already been applied to visualize the arterial chemokine receptor 4 expression in the vasculature, thereby suggesting that this radiotracer may provide such a non-invasive, target-specific read-out in atherosclerotic plaques 14-17. Nonetheless, previous reports on Gallium-labeled PET probes including radiopharmaceuticals targeting prostate-specific membrane antigen- or fibroblast activation protein provided evidence that uptake in calcified vessel segments is rather explained by imaging noise and is not consistently linked to cardiovascular risk 18, 19. Given the increasing application of [^68^Ga]Ga-PentixaFor in oncology 6, 20, 21 and cardiovascular medicine 14-17, 22, 23, we aimed to determine the extent, distribution and frequency of in-vivo vessel wall [^68^Ga]Ga-PentixaFor uptake and to determine its relationship with calcified atherosclerotic plaque (CAP) burden and cardiovascular risk factors (CVRF). In this regard, we also focused on parameters of imaging noise, thereby allowing to determine whether [^68^Ga]Ga-PentixaFor can indeed serve as a surrogate marker of arterial plaque burden.

## Materials and Methods

### General

We investigated 65 oncological patients referred for CXCR4-directed PET/CT in this single-center retrospective analysis. Patients' characteristics are detailed in **Table 1**. Inclusion criteria were available imaging data and clinical information. The presence of the following eight CVRF was recorded for each patient: age above 60 years, male gender, arterial hypertension, hyperlipidemia, diabetes mellitus, history of smoking, body mass index (BMI) above 30, and history of previous cardiovascular events 19. Patients were then stratified based on the numbers of CVRF (0-1 vs 2-3 vs at least 4 19) to identify low, intermediate and high cardiovascular risk groups. Patients with known systemic inflammatory diseases (including vasculitis) and patients who underwent chemotherapy within 4 weeks before imaging were excluded. We also assessed cardiovascular risk using the European Society of Cardiology (ESC) SCORE2/SCORE2-OP risk chart 24, 25. For a subgroup of patients with available FDG-PET/CT within one week of CXCR-directed imaging and no interim treatment changes, FDG-PET/CT data was evaluated in an analogous fashion. The local institutional review board waived the need for further approval, as this was a retrospective analysis (waiver No. #20210726 02). Previous work also analyzed parts of this patient cohort 6, 9, 11, 26-30, but without specifically assessing the vessel wall associated CXCR4 PET-signal.

### Imaging Procedure

As outlined in previous work 19, 29, 31, we carried out PET/CT imaging on a Siemens Biograph mCT 64 or 128 scanner (Siemens Healthineers, Erlangen, Germany). The mean injected activity of [^68^Ga]Ga-PentixaFor was 135.6 MBq (± 22.5 MBq). PET scans were conducted from the top of the skull to the proximal thighs and approximately one hour after radiotracer injection, using the following parameters: iterations, 3; matrix, 200; Gaussian filter, 2.0 mm. Moreover, on the mCT 64 machine, the following settings were applied: 2-3 min/bed position; subsets, 24; point spread function. On the mCT 128 scanner these parameters were used: continuous bed motion at 1.1 mm/s; subsets, 21; time of flight. Low dose CT scans were used for attenuation correction, with activated automatic tube current modulation at a tube voltage of 120 kV, a pitch of 0.8, collimation of 64/128 x 0.6 mm, and reconstructed axial slice thickness ranging from 3.0-5.0 mm.

### Image Analysis

Visual and quantitative analyses of PET, CT and fused PET/CT images were performed on a workstation applying a commercial software package (syngo.via, version VB60A; Siemens Healthineers, Erlangen, Germany). Vascular calcification and high focal radiotracer uptake associated with vessel walls were recorded for seven major artery segments, including the carotid arteries, ascending aorta, aortic arch, descending thoracic aorta, abdominal aorta, iliac arteries, and femoral arteries (paired vessels were considered as one segment) 19. One reader (A.K., board-certified radiologist with ten years of experience in oncological and cardiovascular imaging and 5 years of experience in PET reading) conducted the initial readout, while two board-certified nuclear medicine physicians (S.E.S. and R.A.W.) acted as control read in instances where there was inconclusive vessel wall uptake.

To characterize vessel wall calcification, focal high-density sites in the arterial walls above 130 Hounsfield units (HU) on CT were recorded as CAP 15, 18, 19, 32. For each CAP, we graded the maximum circumferential calcification extent on a 4-point scale: 1, 1-25% of the vessel wall circumference; 2, 26-50% of the vessel wall circumference; 3, 51-75% of the vessel wall circumference; and 4, >75% of the vessel wall circumference 18, 19, 32. Additionally, we measured the maximum CAP thickness in mm perpendicular to the vessel axis.

The background blood-pool standardized uptake value (SUV_mean_) was calculated by averaging values from three distinct regions of interest with a diameter of at least 10 mm on separate slices in the central lumen of the superior vena cava. To evaluate radiotracer uptake associated with the vessel wall, we visually examined axial PET images and fused images with respect to both CAP and the vessel wall 15, 19, 32. Consistent with previous work on PET using [^68^Ga]Ga-Pentixafor and other radiotracers in the context of cardiovascular imaging, we multiplied the background blood-pool uptake for each patient by 1.6, to establish an intra-individual threshold for relevant focal uptake (PentixaFor+) 15, 33-35. We then set the PET SUV window accordingly, to remove all SUV-values below this threshold in the fusion image. Sites of focal uptake associated with the vessel wall of seven major arteries on the resulting image were then assessed: to obtain SUV_max_ of a focal uptake site, we placed a 3-dimensional volume of interest with a minimum diameter of 10 mm to encompass each individual focal uptake site on PET 15, 19. To determine TBR, SUV_max_ of PentixaFor+ sites in the vessel wall was divided by the blood-pool SUV_mean_ 15, 19, 33. For the [^18^F]FDG-PET/CT subgroup, vascular radiotracer uptake was analyzed in an analogous manner 34-37.

To evaluate image noise levels, we utilized coefficient of variation (CoV) measurements within normal liver tissue. By placing a 3 cm region of interest in an area of homogeneous tracer uptake within the unaffected right lobe of the liver, we calculated CoV as the standard deviation ratio to the SUV_mean_ 19, 36.

### Statistical Analysis

We state categorial variables as absolute and relative frequencies and continuous variables as mean ± SD. We tested for normal distribution using the Kolmogorov-Smirnov test, and present normally distributed data as mean ± SD, while median and range are specified otherwise. Correlation analyses were conducted using the Spearman correlation coefficient. Due to overdispersion, negative binomial regression was used to predict the number of PentixaFor+ and/or CT+ lesions by CVRF, age, and gender 18, 19. Linear regression was used to evaluate the relationship between CVRF, age, gender and PentixaFor+ TBR. A *P*-value < 0.05 was considered statistically significant. Analyses were conducted in R (version 4.3.1, R Core Team, 2023) with package MASS (7.3.60) and GraphPad Prism Version 9.3.1 (GraphPad Prism Software, La Jolla, CA, USA) 38, 39.

## Results

### Arterial Wall [^68^Ga]Ga-PentixaFor Uptake

**Table 2** shows comprehensive results for [^68^Ga]Ga-PentixaFor uptake linked to the vessel wall. A total of 1292 focal PentixaFor+ sites associated with major arteries was documented in all 65/65 (100%) patients. The segments with most common wall-associated tracer uptake were the descending thoracic aorta and the abdominal aorta, followed by the iliac arteries. Mean SUV of the venous background was 1.9 ± 0.4 (range, 1.2-3.1), while overall SUV_max_ of PentixaFor+ sites was 4.1 ± 0.4 (range, 2.0-10.5), with highest SUV_max_ seen in the abdominal aorta and the iliac arteries. Overall TBR was 2.3 ± 0.5, ranging from 1.6 to 7.4, with highest values noted in the abdominal aortic segment.

### Arterial Wall Calcification

**Table 3** demonstrates findings of CAP in major arteries. In total, 1431 CAP were documented in 61/65 (93.8%) patients. The most common site of CAP were the iliac arteries and the abdominal aorta, followed by the descending thoracic aorta. The mean scoring for calcification circumference was 1.6 ± 0.9 (range, 1-4), and mean CAP thickness was 2.7 ± 1.1 mm (range, 1-9 mm).

### PentixaFor+ Site Number and TBR do not Correlate with Calcified Plaque Burden

**Figure 1:**
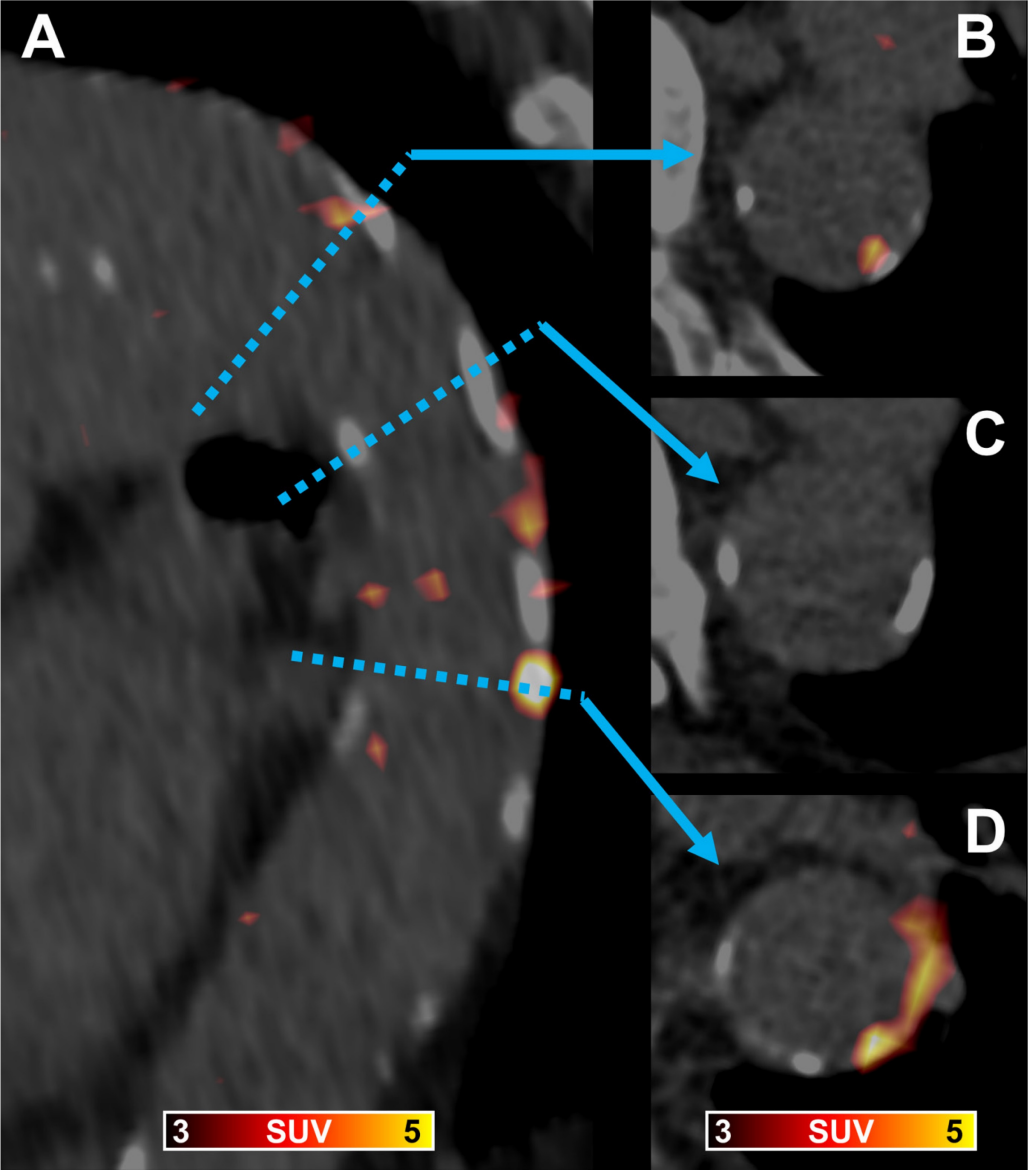
[^68^Ga]Ga-PentixaFor PET/CT in a 89-year old man showing a fused parasagittal image reconstruction of the aortic arch and the descending thoracic aorta (**A**) and three paraaxial slices (**B** trough **D**) at levels indicated by the blue dotted line and arrows. In (**B**) arterial wall radiotracer uptake is partially co-localized with calcification, in (**C**) vessel wall calcification without radiotracer uptake is shown, and in (**D**) vessel wall radiotracer uptake without corresponding calcification and calcification without co-localized radiotracer uptake is visible.

With all patients showing PentixaFor+ sites, 4/65 (6.1%) patients were rated PentixaFor+/CAP-, and the remaining 61/65 (93.8%) PentixaFor+/CAP+ in a per-patient analysis (**Figure 1**).

On a per-segment level, 335/455 segments (73.6%) were PentixaFor+, and 299/455 (65.7%) segments CAP+, with 228/455 (50.1%) PentixaFor+/CAP+ segments, and 107/455 (23.5%) PentixaFor+/CAP- segments. 71/455 (15.6%) segments were PentixaFor-/CAP+, and 49/455 (10.8%) segments were rated negative for both, [^68^Ga]Ga-PentixaFor uptake, and CAP.

On a per-lesion level, 385/1292 (29.8%) PentixaFor+ sites were also CAP+. This amounts to 26.9% (385/1431) of all CAP+ sites. Correlative analyses showed no significant correlations between the number of PentixaFor+ sites or PentixaFor+ site TBR with number of CAP+ sites, calcification circumference score, and CAP thickness (number of PentixaFor+ sites: r ≤ 0.18, *P* ≥ 0.14; PentixaFor+ TBR: r ≤ 0.08, *P* ≥ 0.54).

### CAP Burden-Specific Parameters are Independently Associated with CVRF, while PentixaFor+ Sites and TBR are only Consistently Linked to Image Noise

**Figure 2:**
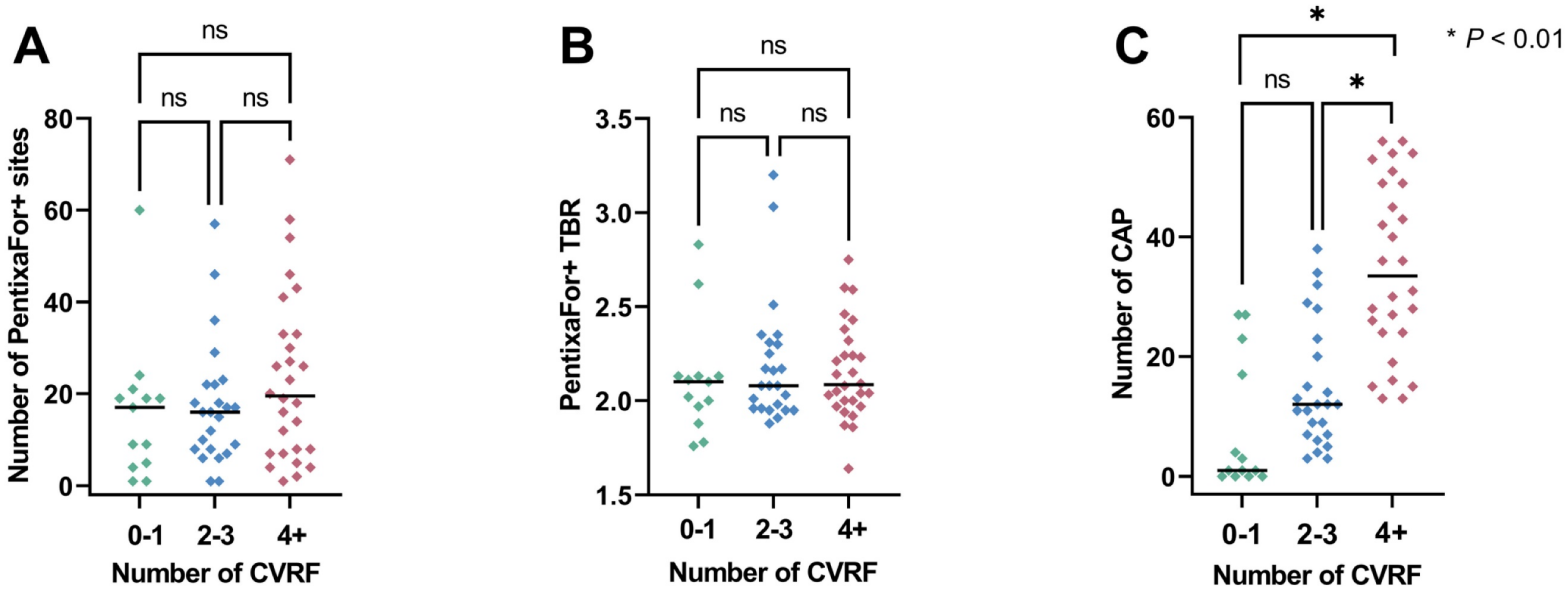
Scatter plots with number of PentixaFor+ sites (**A**), target-to-background ratio (TBR) in those sites (**B**) and the number of calcified atherosclerotic plaques (CAP, **C**). Parameters are separated based on the number of cardiovascular risk factors (CVRF). Median is represented by the horizontal line. Numbers in every subgroup vary based on available PET uptake (number of Pentixafor+ sites and TBR) or CAP sites. ns = not significant

**Figure 3:**
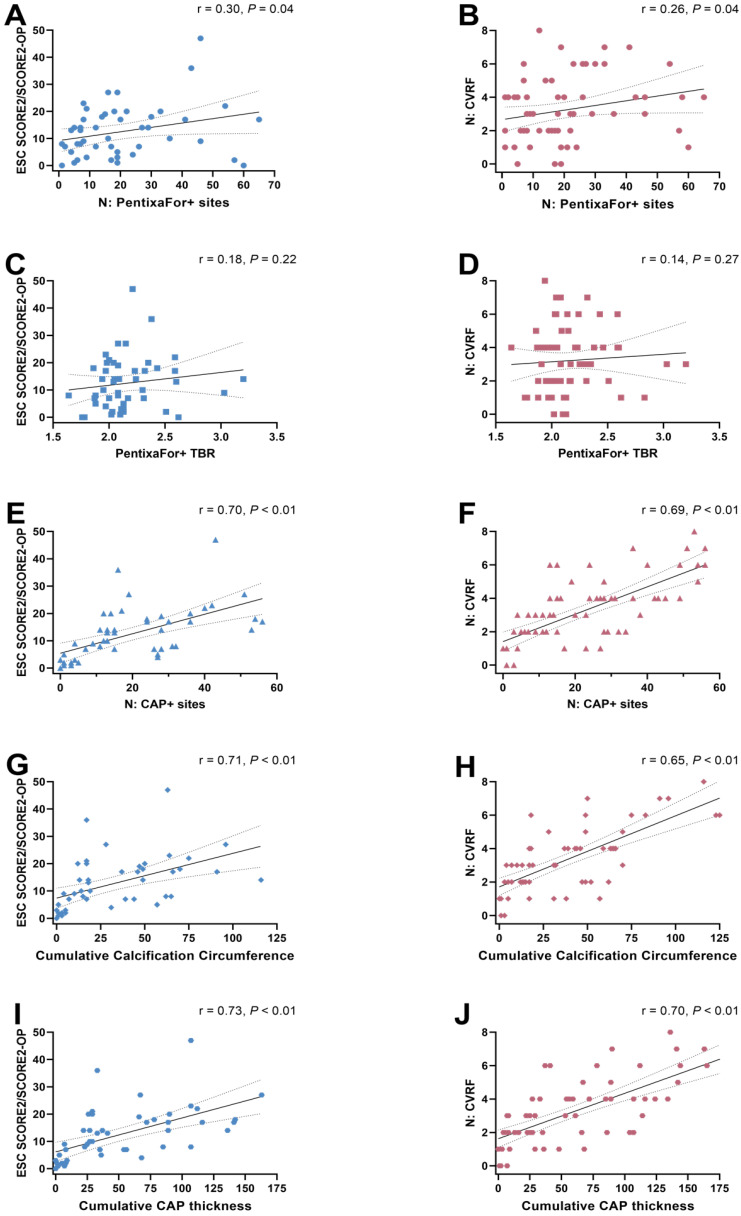
Scatter plots showing associations between the ESC SCORE/SCORE2-OP risk chart (**A, C, E, G, I**) and the number of cardiovascular risk factors (CVRF) (**B, D, F, H, J**) with the number of PentixaFor+ sites (**A, B**), the mean target-to-background-ratio (TBR) in PentixaFor+ sites (**C, D**), the number of calcified atherosclerotic plaques (CAP) (**E, F**), the cumulative calcification circumference (**G, H**), and the cumulative CAP thickness (**I, J**). There is a weak correlation between the number of PentixaFor+ sites and markers of cardiovascular risk (**A, B**), and a strong correlation between indicators of atherosclerotic calcification and cardiovascular risk (**E trough J**).

Overall, the median ESC SCORE2/SCORE2-OP risk chart scoring was 11.5 (range, 0-47) and the median number of CVRF was 3 (range, 0-8). Group-wise analyses provided significant differences only for CAP+ sites, but not for CXCR4+ sites or CXCR4+ TBR (**Figure 2**). The ESC SCORE2/SCORE2-OP risk chart scoring and the number of CVRF correlated significantly with the number of PentixaFor+ sites (r = 0.30 / 0.26, *P* = 0.04, respectively), while there was no significant correlation between both factors and PentixaFor+ site TBR (r ≤ 0.18, *P* ≥ 0.22). For the CAP burden-specific parameters (CAP+ sites, calcification circumference score, CAP thickness), correlative analyses revealed significant associations with the ESC SCORE2/SCORE2-OP risk chart score (r = 0.70 / 0.71 / 0.73; *P* < 0.01, respectively) and the number of CVRF (r = 0.69 / 0.65 / 0.70; *P* < 0.01, respectively (**Figure 3**)).

Regression analysis results are summarized in **Table 4**. Univariable regression analysis revealed significant associations between the number of PentixaFor+ sites and BMI (odds ratio [OR] 1.08; 95% confidence interval [CI], 1.02-1.14; *P* < 0.01), injected activity (OR 0.99, CI 0.99-1.00, *P* = 0.04), and CoV (OR 1.07, CI 1.05-1.10, *P* < 0.01). In a multivariable model, only injected activity (OR 1.00, CI 0.99-1.00, *P* < 0.01), and CoV (OR 1.06, CI 1.06-1.07, *P* < 0.01) remained significant predictors of the PentixaFor+ site number. For PentixaFor+ site TBR, BMI (OR 1.03, CI 1.02-1.05, *P* < 0.01), and CoV (OR 1.02, CI 1.01-1.03, *P* < 0.01) showed significant associations in univariable regression, while only CoV (OR 1.02, CI 1.01-1.03, *P* < 0.01) remained significant in the multivariable model.

Unlike results for PentixaFor+ sites, the number of CAP+ sites were significantly associated in univariable analysis with the CVRF arterial hypertension (OR 2.32, CI 1.52-3.51, *P* < 0.01), history of previous cardiovascular events (OR 1.87, CI 1.08-3.47, *P* = 0.03), gender (OR 1.81, CI 1.12-2.86, *P* = 0.01), smoking (OR 1.64, CI 1.05-2.57, *P* = 0.03) and age (OR 1.06, CI 1.04-1.08, *P* < 0.01). All of these factors remained significant in multivariable analysis (*P* < 0.01, respectively): gender (OR 1.56, CI 1.36-1.78), arterial hypertension (OR 1.49, CI 1.29-1.74), smoking (OR 1.27, CI 1.14-1.43), and history of previous cardiovascular events (OR 1.22, CI 1.07-1.38), and age (OR 1.03, CI 1.02-1.04).

### CXCR4-directed CoV Correlates with BMI, Injected Activity, Number of PentixaFor+ Sites and TBR

**Figure 4:**
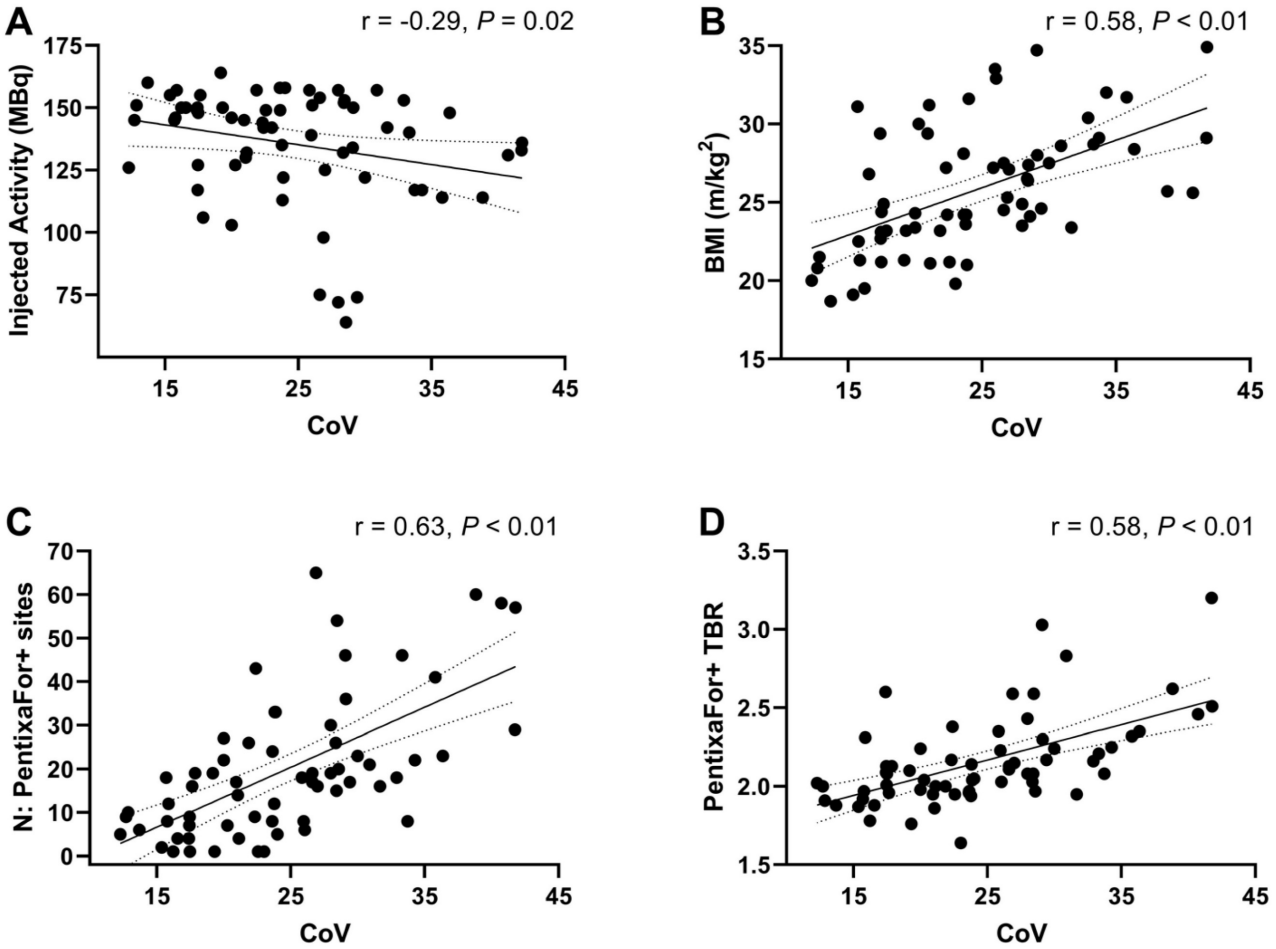
Scatter plots demonstrating associations between image noise determined by coefficient of variation (CoV) within normal liver parenchyma and injected activity (**A**), body mass index (BMI) (**B**), the number of PentixaFor+ sites (**C**), and PentixaFor+ site target-to-background ratio (TBR) (**D**). Lower injected activities and higher BMI were associated with higher image noise. In addition, more PentixaFor+ sites and higher TBR were associated with higher levels of image noise.

On CXCR4-directed imaging, image noise levels determined by CoV measurement within normal liver parenchyma showed a mean CoV of 24.5 ± 7.4. Image noise correlated significantly with injected activity (r = -0.29, *P* = 0.02) and BMI (r = 0.58, *P* < 0.01). In addition, significant correlations were noted between CoV and the number of PentixaFor+ sites (r = 0.63), and PentixaFor+ site TBR (r = 0.58; *P* < 0.01, respectively; **Figure 4**). For [^18^F]FDG-PET/CT, a mean CoV of 13.3 ± 2.2 was noted, and no significant correlations with BMI, number of focal uptake sites or site TBR was observed (r ≤ 0.25; P ≥ 0.18).

### Arterial Wall [^18^F]FDG Uptake

In a subgroup of 32/65 (49.2%) patients, data from [^18^F]FDG-PET/CT imaging within 1 week of CXCR4-directed imaging was available. Of those, 26/32 (81.3%) showed relevant focal uptake associated with the vessel wall (FDG+) in 201 sites. **Supplemental Table 1** details vascular [^18^F]FDG uptake. Of 224 analyzed vessel segments, 79 (35.3%) were FDG+, with 62/224 (27.7%) FDG+/CAP+, and 17/224 (7.6%) FDG+/CAP- segments. 96/224 (42.9%) segments were FDG-/CAP+, and 49/224 (21.9%) segments did not show relevant tracer uptake or calcification. Of 201 FDG+ sites, 99 (49.3%) showed concomitant calcification, which translates to 13.5% (99/731) of all CAP+ sites. There were no significant correlations between number of FDG+ sites or FDG+ TBR and number of CAP+ sites, calcification circumference score, and CAP thickness (number of FDG+ sites: r ≤ 0.29, P ≥ 0.11; FDG+ TBR: r ≤ 0.36 P ≥ 0.07). FGD+ TBR correlated significantly with the number of CVRF (r = -0.45, *P* = 0.02), but not with the ESC SCORE2/SCORE2-OP risk chart score (r = -0.22, *P* = 0.36). There were no significant correlations between number of CVRF or ESC SCORE2/SCORE2-OP risk chart score and number of FDG+ sites (r ≤ 0.32, P ≥ 0.12).

Compared to CXCR-directed imaging, the number of FDG+ sites showed a significant correlation with the number of PentixaFor+ sites (r = 0.36, *P* = 0.04), while FDG+ TBR and PentixaFor+ TBR showed no correlation (r = 0.15, *P* = 0.46).

## Discussion

In 65 oncologic patients who underwent [^68^Ga]Ga-PentixaFor PET/CT, high focal uptake in major arteries was observed in 1292 sites in all patients (100%). Simultaneously, 1431 sites of vessel wall calcification were documented in 61/65 (93.8%) subjects, with concurrent tracer uptake in less than one third, thereby raising the question whether observed uptake in plaques is a reliable surrogate marker of cardiovascular risk. Our analyses showed no association of vessel wall tracer uptake and varying parameters of CT-based vascular calcification. However, we observed a weak correlation between the number of PentixaFor+ sites and ESC SCORE2/SCORE2-OP chart and the number of CVRF. Uni- and multivariable regression models, however, identified no consistent link between vessel wall uptake and CVRF, but with CoV, suggesting that observed radiotracer accumulation in the arterial tree may be explained by image noise. Those findings were supported by substantial correlative indices between CoV and number and intensity of [^68^Ga]Ga-PentixaFor uptake sites. Of note, serving as reference, CAP burden provided a consistent and independent link with varying indicators of cardiovascular risk, including hypertension, smoking or history of cardiovascular events. Taken together, relative to CT-based assessment of CAP, [^68^Ga]Ga-PentixaFor PET may be rather less suitable as an image biomarker in atherosclerosis. In contrast, [^18^F]FDG PET showed a considerably lower CoV. CoV also did not exhibit any relevant correlations with the number of FDG+ vessel wall uptake sites and TBR. These findings suggest that image noise has a lesser impact on cardiovascular [^18^F]FDG PET imaging.

Arterial damage activates varying cell types involved in the CXCR4/CXCL12 axis, which all contribute to an inflammatory phenotype in atherosclerosis, e.g., macrophages, neutrophiles, or B-cells 12, 40. By exerting atheroprotective effects, CXCR4 modulators may provide a therapeutic option in patients affected with atherosclerosis 12. Nonetheless, such chemokine receptor-interacting drugs should be used with caution in the context of cardiovascular medicine, as this receptor subtype also mediates stem cell mobilization of the bone marrow and is involved in trafficking of stem cell progenitor cells 41. In this regard, previous reports have already reported on high [^68^Ga]Ga-PentixaFor uptake in CAP+ sites, which even outperformed other inflammatory-targeting radiotracers such as [^18^F]FDG. For instance, Kircher et al. reported on substantial correlative indices between CT-based HU and [^68^Ga]Ga-PentixaFor TBR, which was even more pronounced when compared to [^18^F]FDG-based vessel wall quantification 15. Conducting a more thorough CT-based assessment including the number of CAP sites, calcification circumference and thickness, we could not establish such an association with PentixaFor+ sites and TBR for any of those CAP burden-reflecting parameters. Relative to Kircher and colleagues 15, we also conducted uni- and multivariable regression analyses to determine an independent association between CVRF and CXCR4 PET vessel wall uptake. Of note, both PentixaFor+ sites and TBR failed on multivariable analyses, while CAP+ sites were independently associated with varying factors of cardiovascular risk (**Table 4**). These findings are in line with Weiberg et al., who also investigated CAP burden and visual and quantitative [^68^Ga]Ga-PentixaFor uptake in the arterial tree. Comparable to our findings, they also reported only on independent associations between CT-based atherosclerotic plaques and age and previous cardiovascular events 32. Of note, recent analyses on other 68Ga-labelled radiotracers targeting fibroblasts or prostate specific membrane antigen also demonstrated high TBR in the vessel walls, indicative for excellent image contrast 18, 19. Further in-depth analyses, however, then revealed that arterial uptake was linked to CoV, injected activity and/or BMI, suggesting that the observed uptake in the vasculature was most likely explained by image noise 18, 19. Therefore, we also aimed to conduct such an inherent quality control study to define whether [^68^Ga]Ga-PentixaFor may indeed serve as a potential imaging biomarker for patients affected with atherosclerosis. Similar to other PET studies on 68-Gallium 19, only CoV was consistently linked to the numbers of PentixaFor+ sites and PentixaFor+ TBR (**Table 4**). Thus, despite weak correlations between CVRF and ESC SCORE2/SCORE2-OP in simple regression analyses (**Figure 3**), the herein provided results indicate that the quantitative and visual uptake in the vessels is rather explained by image noise, most likely due to the underlying 68-Gallium radiochemistry. Of note, the reference radiotracer [^18^F]FDG reflecting macrophages has also been extensively investigated to determine atherosclerotic activity 42. In our analysis, CoV did not provide any relevant associations with the number of FDG+ vessel wall uptake sites and TBR, indicative for less impact of image noise on cardiovascular [18F]FDG PET imaging. As such, those findings may provide evidence that fluorine radiochemistry may overcome physical drawbacks related to the use of 68-Gallium, including decreased positron yield or increased positron range causing increased partial volume effect 43. In this regard, also similar to previous work investigating ^68^Ga-labelled PET agents 19, we also observed a substantial correlation between CoV and PentixaFor+ TBR 19 - a phenomenon that may be explained by activity clustering caused by PET reconstruction algorithms 18. As such, these challenges posed by the 68-Gallium radiochemistry could be addressed by developing novel 18F-labeled radiotracers that target chemokine receptors 44, or by the broader use of total-body PET scanners. These scanners offer greatly enhanced sensitivity and resolution, e.g. enabling ultra-late imaging after several half-lives of the used radionuclide 36. In a study by Derlin and colleagues on an ultra-sensitive total-body PET scanner, signal in the vessel wall showed a marked increase over time, while the blood-pool signal gradually decreased 36. The subsequent improvement of TBR and contrast thus potentially alleviates some drawbacks from unfavorable 68-Gallium radiochemistry, especially regarding uptake in vessel walls 36. Also, since an inverse relationship between the amount of injected activity and image noise has been shown for ^68^Ga-based tracers, the use of higher injected activities should be considered, especially in the context of cardiovascular research 18. The utilization of novel noise-reducing reconstruction algorithms may prove beneficial, particularly in the context of investigations that are susceptible to image noise, such as the assessment of vessel-wall tracer uptake 45.

The results of our study are limited by several factors. First, the retrospective design and the small number of patients must be considered, as this limits the study's ability to establish causation and introduces potential biases in patient selection. A prospective study would provide stronger evidence. Second, the study focused on oncological patients, thus limiting the generalizability of findings to the broader populations, especially those with known cardiovascular disease. Third, as a retrospective analysis of cancer patients, definitive cardiovascular endpoints have not been documented. Forth, as also proven earlier 16, this study lacks a direct comparison of PET findings with histopathology or animal tissue, which would have strengthened the power of the study. Fifth, a factor causing more pronounced partial volume effects and thus image noise is the high positron range of ^68^Ga compared to ^18^F, which may hamper correct localization of radionuclide signal originating in the vessel wall 19, 32. Last, the lack of positive findings concerning a relationship between tracer uptake and cardiovascular risk may also be due to small vessel wall diameters below spatial resolution capacity of the applied PET systems. Consequently, this may also contribute to partial volume effects and result in image noise 19, 32, 35. Those issues, however, may be addressed by novel, ^18^F-labelled chemokine receptor-targeting radiotracers 44 or by a more widespread adoption of total-body PET scanners, which provide improved sensitivity and resolution, potentially mitigating this concern, particularly in the context of vessel wall uptake 36.

## Conclusion

In oncologic patients undergoing [^68^Ga]Ga-PentixaFor PET, high focal tracer uptake in the arterial vessel wall was documented at 1292 sites in 65/65 (100%) of patients. There was no significant association between arterial uptake and calcification as a morphological indicator of cardiovascular risk. Although we observed a weak correlation between the number of PentixaFor+ sites and the number of CVRF and ESC SCORE2/SCORE2-OP risk scoring, regression analyses showed no consistent link between vessel wall uptake and CVRF, but with morphological CAP burden. PET-based findings of apparent vessel wall uptake, however, were most likely due to image noise.

## Supplementary Material

Supplementary table.

## Figures and Tables

**Table 1:** Patients' characteristics (n = 65).

Age^†^	63 ± 13 years (25 - 88)
**Gender (female)^‡^**	22/65 (33.8)
**BMI (kg/m^2^)^†^**	25.8 ± 4.0 (18.7 - 34.9)
**Tumor entity^‡^**	
NEN	21/65 (32.3)
Lung (SCLC and NSCLC)	14/65 (21.5)
HNSCC	12/65 (18.5)
Pancreas Adenocarcinoma	5/65 (7.7)
HCC	4/65 (6.2)
Other*	9/65 (13.8)
**Cardiovascular risk factors^‡^**	
Male gender	43/65 (66.2)
Age > 60 years	43/65 (66.2)
Hypertension	38/65 (58.5)
History of smoking	28/65 (43.1)
Hypercholesterinemia	14/65 (21.5)
Prior cardiovascular event	11/65 (16.2)
BMI > 30 kg/m²	11/65 (16.2)
Diabetes mellitus	10/65 (15.4)

^†^ Values are mean ± standard deviation, with range in parenthesis. ^‡^ Values are numbers of patients, with percentages in parenthesis. *including sarcoma (n = 3), cholangiocellular carcinoma (n = 1), mesothelioma (n = 1), ovarial carcinoma (n = 1), renal cell carcinoma (n = 1), desmoplastic small round cell tumor (n = 1), granulosa cell tumor (n = 1). BMI = body mass index, NEN = neuroendocrine neoplasm, SCLC = small cell lung carcinoma, NSCLC = non small cell lung cell carcinoma, HNSCC = head and neck squamous cell carcinoma, HCC = hepatocellular carcinoma.

**Table 2:** Arterial wall [^68^Ga]Ga-PentixaFor uptake

	Carotid arteries	Ascending aorta	Aortic arch	Descending thoracic aorta	Abdominal aorta	Iliac arteries	Femoral arteries	All vessels
Number (%) of patients with vessel wall associated [^68^Ga]Ga-PentixaFor uptake	33 (50.8)	42 (64.6)	50 (76.9)	63 (96.9)	59 (90.8)	50 (76.9)	43 (66.2)	65 (100)
Total number of uptake sites	60	86	113	365	286	218	164	1292
Sites with concomitant calcification n (%)	14 (23.3)	1 (1.2)	33 (28.3)	62 (17.0)	142 (49.7)	93 (42.7)	41 (25.0)	385 (29.8)
SUV_max_								
Mean ± SD	3.7 ± 0.9	4.0 ± 1.0	4.0 ± 1.1	4.1 ± 1.1	4.2 ± 1.2	4.2 ± 1.2	3.9 ± 0.9	4.1 ± 1.1
Range	2.0-5.2	2.5-7.3	2.3-9.3	2.1-9.1	2.0-10.5	2.1-8.8	2.2-6.5	2.0-10.5
TBR								
Mean ± SD	2.2 ± 0.4	2.3 ± 0.4	2.3 ± 0.5	2.3 ± 0.5	2.4 ± 0.6	2.3 ± 0.5	2.2 ± 0.4	2.3 ± 0.5
Range	1.6-3.2	1.8-3.7	1.6-4.3	1.6-5.7	1.6-7.4	1.6-4.5	1.6 - 4.4	1.6-7.4
SUV_mean blood-pool_								
Mean ± SD								1.9 ± 0.4
Range								1.2-3.1

Suv; TBR = target-to-background ratio; SUV_mean blood pool_ = mean standardized uptake value calculated by averaging values from three distinct regions of interest with a diameter of at least 10 mm on separate slices in the central lumen of the superior vena cava providing the reference to calculate TBR.

**Table 3:** Arterial wall calcification

	Carotid arteries	Ascending aorta	Aortic arch	Descending thoracic aorta	Abdominal aorta	Iliac arteries	Femoral arteries	All vessels
Number (%) of patients with arterial wall calcification	38 (58.5)	10 (15.8)	48 (73.8)	42 (64.6)	55 (84.6)	58 (89.2)	44 (67.7)	61 (93.8)
Total number of calcification sites	82	12	132	267	345	390	203	1431
Calcification circumference score								
Mean ± SD	2.0 ± 1.0	1.0 ± 0.0	1.1 ± 0.3	1.1 ± 0.3	1.8 ± 1.1	1.8 ± 1.0	1.8 ± 1.0	1.6 ± 0.9
Range	1-4	1-1	1-3	1-3	1-4	1-4	1-4	1-4
Calcification thickness (mm)								
Mean ± SD	2.4 ± 1.1	2.2 ± 0.8	3.0 ± 1.4	2.3 ± 0.8	2.7 ± 1.1	2.9 ± 1.2	2.5 ± 1.0	2.7 ± 1.1
Range	1-5	1-4	1-9	1-6	1-7	1-7	1-5	1-9

**Table 4:** Factors associated with numbers of PentixaFor+ sites, PentixaFor+ TBR, and numbers of CAP+ sites

	N: PentixaFor+ sites							PentixaFor+ TBR								N: CAP+ sites						
**Factor**	Univariable				Multivariable			Univariable				Multivariable				Univariable			Multivariable			
	OR	95% CI	*P*	OR		95% CI	*P*		OR	95% CI	*P*	OR	95% CI	*P*	OR		95% CI	*P*		OR	95% CI	*P*
Gender (male)	1.39	0.90-2.11	0.13						1.01	0.87-1.17	0.87				1.81		1.12-2.86	0.01		**1.56**	**1.36-1.78**	**<0.01**
Age	1.01	0.99-1.02	0.31						1.00	0.99-1.01	0.92				1.06		1.04-1.08	<0.01		**1.03**	**1.02-1.04**	**<0.01**
Smoking	1.22	0.81-1.85	0.34						1.07	0.93-1.24	0.32				1.64		1.05-2.57	0.03		**1.27**	**1.14-1.43**	**<0.01**
Diabetes	1.12	0.66-2.03	0.70						1.11	0.91-1.34	0.32				1.66		0.93-3.20	0.11				
Hyper-lipidemia	1.45	0.92-2.37	0.12						1.16	0.99-1.36	0.08				1.50		0.90-2.61	0.13				
Hyper-tonus	1.09	0.72-1.64	0.68						0.91	0.79-10.5	0.21				2.32		1.52-3.51	<0.01		**1.49**	**1.29-1.74**	**<0.01**
Previous CV event	1.24	0.74-2.18	0.44						0.97	0.80-1.17	0.75				1.87		1.08-3.47	0.03		**1.22**	**1.07-1.38**	**<0.01**
BMI	1.08	1.02-1.14	<0.01	1.00		0.98-1.02	1.00		1.03	1.02-1.05	<0.01	1.01	1.00-1.03	0.11	1.00		0.95-1.07	0.88				
Injected activity	0.99	0.98-1.00	0.04	**1.00**		**0.99-1.00**	**<0.01**		1.00	1.00-1.00	0.52				0.99		0.98-1.00	0.14				
CoV	1.07	1.05-1.10	<0.01	**1.06**		**1.06-1.07**	**<0.01**		1.02	1.01-1.03	<0.01	**1.02**	**1.01-1.03**	**<0.01**	1.00		0.97-1.04	0.80				
																						

TBR = target-to-background ratio, CAP = calcified atherosclerotic plaque, OR = Odds ratio, CI = confidence interval, BMI = body mass index, CoV = coefficient of variation; in bold are factors with significant associations in multivariable analyses.

## Abbreviations

BMI: body mass index
CAP: calcified atherosclerotic plaque burden
CI: confidence interval
CoV: coefficient of variation
CVRF: cardiovascular risk factors
CXCR4: C-X-C motif chemokine receptor 4
ESC: European Society of Cardiology
HU: Hounsfield units
OR: odds ratio
SUV: standardized uptake value
TBR: target to background ratio
