# Supplementary material for: C-X-C motif chemokine receptor 4-directed PET signal in the arterial tree is not consistently linked to calcified plaque burden and cardiovascular risk
Source: Theranostics. 2025 Jan 1;15(3):804–14. doi: 10.7150/thno.102910 (PMC11700869; doi:10.7150/thno.102910)
Supplement: Supplementary file 1 — Supplementary table. [file thnov15p0804s1.pdf]

Supplemental Table 1. Arterial wall [^{18}F]FDG uptake

	Carotid arteries	Ascending aorta	Aortic arch	Descending thoracic aorta	Abdominal aorta	Iliac arteries	Femoral arteries	All vessels
Number (%) of patients with vessel wall associated [^{18}F]FDG uptake	6 (18.8)	11 (34.4)	10 (31.3)	17 (53.1)	18 (56.3)	10 (31.3)	5 (15.6)	26 (81.3)
Total number of uptake sites	12	16	21	61	59	22	10	201
Sites with concomitant calcification n (%)	10 (83.3)	2 (12.5)	8 (38.1)	23 (37.7)	33 (55.9)	16 (72.7)	7 (70.0)	99 (49.5)
SUV_{max}								
Mean ± SD	3.3 ± 0.5	3.3 ± 0.9	3.7 ± 0.8	3.7 ± 0.7	3.8 ± 0.9	3.6 ± 0.9	3.1 ± 0.7	3.6 ± 0.8
Range	2.3-4.0	2.1-5.4	2.2-5.1	1.9-5.0	1.8-5.8	2.3-5.5	2.1-4.0	1.8-5.8
TBR								
Mean ± SD	1.8 ± 0.2	2.0 ± 0.2	2.0 ± 0.2	2.0 ± 0.2	2. 0± 0.3	1.9 ± 0.3	1.8 ± 0.1	2.0 ± 0.3
Range	1.6-2.2	1.8-2.7	1.6-2.5	1.6-2.9	1.6-2.9	1.6-2.5	1.7 – 2.0	1.6-2.9
$SUV_{mean\ blood\text{-}pool}$								
Mean ± SD								1.8 ± 0.3
Range								1.1-2.4

SUV = standardized uptake value; TBR = target-to-background ratio; $SUV_{mean\ blood\ pool}$ = mean standardized uptake value calculated by averaging values from three distinct regions of interest with a diameter of at least 10 mm on separate slices in the central lumen of the superior vena cava providing the reference to calculate TBR.
